# Sensitive Marker of the Cisplatin-DNA Interaction: X-Ray Photoelectron Spectroscopy of CL

**DOI:** 10.1155/2012/649640

**Published:** 2012-10-24

**Authors:** Fangxing Xiao, Xiaobin Yao, Qianhong Bao, Danzhen Li, Yi Zheng

**Affiliations:** State Key Laboratory Breeding Base of Photocatalysis, Research Institute of Photocatalysis, College of Chemistry and Chemical Engineering, Fuzhou University, Fuzhou 350002, China

## Abstract

The development of cisplatin and Pt-based analogues anticancer agents requires knowledge concerning the molecular mechanisms of interaction between such drugs with DNA. However, the binding dynamics and kinetics of cisplatin reactions with DNA determined by traditional approaches are far from satisfactory. In this study, a typical 20-base oligonucleotide (C**G**T**G**ACA**G**TTATT**G**CA**GG**C**G**), as a simplified model representing DNA, was mixed with cisplatin in different molar ratios and incubation time. High-resolution XPS spectra of the core elements C, N, O, P, and Cl were recorded to explore the interaction between cisplatin and DNA. From deconvoluted Cl spectra we could readily differentiate the covalently bound chlorine from ionic chloride species in the cisplatin-oligo complexes, which displayed distinct features at various reaction times and ratios. Monitoring the magnitude and energy of the photoelectron Cl 2p signal by XPS could act as a sensitive marker to probe the interaction dynamics of chemical bonds in the reaction of cisplatin with DNA. At 37°C, the optimum incubation time to obtain a stable cisplatin-oligo complex lies around 20 hrs. This novel analysis technique could have valuable implications to understand the fundamental mechanism of cisplatin cytotoxicity and determine the efficiency of the bonds in treated cancer cells.

## 1. Introduction

*Cis*-diamminedichloroplatinum (cisplatin) has become the most frequently used drugs in the chemotherapy treatment of most malignant cancers [1–4]. The enhancement of DNA damage by cisplatin in concomitant chemoradiation therapy further stresses the application of the cisplatin analogues drug in cancer treatment [5, 6]. Cisplatin kills cancer cells via apoptosis caused by its binding and cross-linking to nuclear DNA, in which different intermediate adducts formed when cisplatin binds to DNA are thought to be responsible for its cytotoxicity. Therefore, probing the molecular basis of such underlying interactions could have significant implications for the optimum clinical application of cisplatin and platinum-based antitumor drugs.

The process of Pt binding to nucleobases involves complex pathways which are kinetically controlled, rendering accurate identification of the reaction rates and products rather difficult. In this regard, a myriad of analyzing technologies to date have been harnessed to investigate the manifold of adducts from the cisplatin—DNA reactions, including ionic exchanged chromatography [7], capillary electrophoresis (CE) [8–11], high-performance liquid (HPLC) [12, 13], X-ray diffraction [14–18], NMR spectroscopy [12, 19–21], extended X-ray absorption fine structure (EXAFS), Fourier transform infrared (FTIR) [22], a collection of mass spectroscopy (MS) techniques [8, 16, 17, 23–32], and time-resolved femtosecond laser spectroscopy [33]. These series of combinatorial studies have revealed that the reaction initiates predominantly from the aquation process of cisplatin to the monofunctional and bifunctional adducts on the time scale of days to weeks. The major configurations are the GpG and ApG intrastrand adducts that together account for 80–90% of the bound Pt. Despite the above joint techniques used for the determination of the products deriving from cisplatin binding to DNA, the challenge for elucidating the intermediate binding mechanism has met with limited success. Meanwhile, many other factors such as DNA sequence types and buffer can also affect the observed adduct profile. Hence, the interaction of cisplatin with DNA at the molecular scale was found to be fairly intricate in addition to its significance in cancer therapy. In practice, the application of Pt-based chemotherapeutic drugs requires a simple and direct method to monitor such interaction process. 

Previous studies revealed the cisplatin aquation and subsequent reactions to be mainly oriented toward the Pt binding process. Nonetheless, important information regarding the chloro ligands released from cisplatin is generally neglected because it is difficult to obtain *via* conventional HPLC-based techniques. Moreover, reports on the interaction of cisplatin with DNA, by monitoring the chlorine signal with X-ray photoelectron spectroscopy (XPS), have not yet been reported. In the present study, new insights into the interaction of cisplatin with DNA are obtained by monitoring chlorine instead of the more conventional Pt signal with XPS. XPS is one of the most powerful techniques of chemical characterization. Compared to traditional analytical methods mentioned above, not only does it allow quantitative elemental analysis of various sample films, but also, more significantly, provides relatively precise information with regard to the modification of chemical bonds and elemental chemical state.

 XPS characterization of cisplatin-DNA interaction has not been reported apart from the tentative work performed by Millard et al. in 1975 [34]. Depending on the sensitive binding energy (BE) shift of DNA constituent elements, including N 1s, O 1s, and P 2p, the authors concluded that the cisplatin—DNA reaction was initiated by the attack of Pt at the N7 position and neighboring O6 sites of guanine (G). Compared to the result of other techniques, which showed that cisplatin preferentially bonds to N7 sites of purine [4, 14, 15, 35], it thus appears highly desirable to systematically compare the cisplatin—DNA to pure DNA by XPS again with current improved sensitivity and resolution. *With respect to the discrimination between aqua and chloroligands XPS is particularly well suited to detect the chemical changes of chlorine, which would be a powerful signal to monitor the kinetic interaction between cisplatin and DNA.*

Herein, in order to simply elucidate the general chemistry scenario of cisplatin and DNA, a typical 20-base oligonucleotide, that is, the “oligo,” C**G**T**G**ACA**G**TTATT**G**CA**GG**C**G**, with 8 sites of G is devised and used as a proof-of-concept model to represent cellular DNA. Cisplatin-oligo complexes are prepared with different ratios of cisplatin and reaction time at 37°C, as it does in most *in vitro* reactions [36, 37]. High-resolution spectra of the principal elements of oligonucleotide (C, N, O, P and Cl) with and without binding to cisplatin are accurately measured by XPS to probe the detailed chemical bond transformation of oligo in the dynamic process of cisplatin binding to DNA as compared with pure DNA. The significant differences of high-resolution spectra of Cl demonstrate that monitoring the chloro ligand by XPS could provide an easily accessible and effective way to disclose the detailed reaction dynamics of cisplatin. More significantly, the technique may be extended to other Pt-relating anticancer drugs used in chemotherapy. We also specifically discuss for the first time the compositional alterations of DNA in relation to its binding with cisplatin. It is hoped that our current work could motivate explorations focused on the mechanical analysis between platinum-based antitumor drugs and DNA with XPS. 

## 2. Materials and Methods

### 2.1. Materials

The HPLC purified 20-mer oligo (C**G**T**G**ACA**G**TTATT**G**CA**GG**C**G**, molecular weight 6063) was purchased from Invitrogen. The amount of oligo was determined by measuring its UV absorption at 260 nm of 28.5 *μ*g/OD provided by Invitrogen. *Cis-*diammineplatinum (II) dichloride (cisplatin) was obtained from Sigma Aldrich and used without any further purification. The tantalum substrates (Alfa Aesar, 99.95%) were cleaned by ultrasonic with ethanol and deionized water (ddH_2_O, Milipore, 18.2 MΩ·cm resistivity) for three times and dried in glove box at room temperature prior to each deposition of DNA.

### 2.2. Preparation of Oligo and Cisplatin-Oligo Films

10 *μ*L of oligo solution (100 *μ*M) were deposited on the tantalum foil forming a drop with 2 mm radius and dried in glove box under nitrogen atmosphere at room temperature. Assuming a uniform distribution of the oligo on the substrate and a density of 1.7 g cm^−3^, the thickness of the film was estimated to be 50 molecular layers (ML).

0.1 mg of cisplatin was dissolved in 500 *μ*L dd H_2_O at 55°C for 30 minutes to obtain the cisplatin solution. Subsequently, the cisplatin solution was mixed with oligos to prepare the cisplatin-oligo complexes with different ratios (*R*_*i*_), where *R*_*i*_ is defined as the molar ratio of platinum atom to oligo molecule. In brief, 5.2 *μ*L of 100 *μ*M oligo were mixed with 1.56 *μ*L of cisplatin with different concentration (from 100 to 2000 ng/*μ*L) to get the final cisplatin-oligo complexes with molar ratios of 1 : 1, 2 : 1, 4 : 1, 8 : 1, 10 : 1, 12 : 1, 20 : 1, respectively. The cisplatin-oligo complexes were simultaneously incubated at 37°C in the dark for 4 h in PCR (GeneAmp PCR System 9700). Finally, the aqueous cisplatin-oligo solution was deposited on a tantalum substrate and dried in glove box with nitrogen atmosphere at room temperature to form the films of cisplatin-oligo complexes with different ratios. With the same amount of oligos in the complexes the thickness of all the cisplatin-oligo films was estimated to be 26 ML. 

For the experiment of various incubation times, similarly, cisplatin-oligo complexes with ratio of 8 : 1 were incubated at 37°C from 0 to 24 h with 4 h time interrupt in between. Each sample was immediately removed from PCR at given time and formed the corresponding films for further XPS analysis. 

### 2.3. XPS Measurement

XPS measurements were conducted using a commercial XPS system (Thermo Scientific ESCALAB 250) equipped with a dual anode X-ray gun, a concentric hemispherical electron energy analyzer, and a magnetic electron lens. The apparatus was operated with a monochromatic Al K*α*  beam as the excitation source (*hυ* = 1486.6 eV) with the energy resolution of 0.45 eV. The emission current was kept at 6 mA under a base pressure of  3.8 × 10^−10^ mbar. The XPS spot size and analyzer field were below 1 mm^2^. The neutralizing electron gun was turned on in the low energy mode with emission current of 100 mA to eliminate the charging of the samples during X-ray irradiation. Before the measurements each film was first etched by Argon ion operated at 2 KV and 1 *μ*A for 30 s. The material removed was estimated to be approximately 1.2 nm (0.4 Å/s × 30 s = 1.2 nm). No difference is observed in the P 2p spectra before and after Ar etching, while three monitored spots are randomly chosen on each sample surface (See Supporting Information Figure S1 in Supplementary Material available online at doi:10.1155/2012/649640). It indicates that the applied Ar etching does not affect the chemical composition of oligo and oligo-Pt complex on the Ta substrate under the present conditions. Instead it can helpfully remove surface contaminants and expose the fresh sample particularly for C, O, and N spectra. Since the XPS probing length is approximately 10 nm (3–5 ML) on a sample surface [38], the thickness of oligo and cisplatin oligo films with profile of 50 and 26 ML in the present study is found to be sufficient for the XPS characterization. 

The hemispherical electron energy analyzer input axis was normal to the sample surface. XPS survey spectra from BE of 0 to 1200 eV were recorded in the fixed analyzer transmission mode with a pass energy of 100 eV and energy steps of 1 eV. The typical peaks of elements, C 1s, N 1s, O 1s, P 2p, Cl 2p, and Pt 4f, were recorded separately with pass energy of 50 eV and energy steps of 0.05 eV. The energy scale of XPS spectra was calibrated according to the standard C 1s BE line of 285.0 eV, which corresponds to standard hydrocarbon energy of C–H and C–C bonds. The work function of the system was 4.38 eV.

### 2.4. XPS Peak Analysis

Commercial XPS analysis software (Advantage 4.37) was used to calculate the peak area and deconvolute the peaks of high-resolution spectra. The atomic ratios of composition elements in the film were calculated according to the corresponding fitted peak area and corrected by the instrument sensitivity factors (SF), leading to the more reliable result with error of 5%. The convolution of Lorentzian and Gaussian line shapes were employed to fit the core-level spectra of individual peaks as well as the Shirley function to model the background. 

## 3. Results and Discussion

### 3.1. The Reaction Process of Cisplatin with Oligos during the Incubation Time

**Figure 1:**
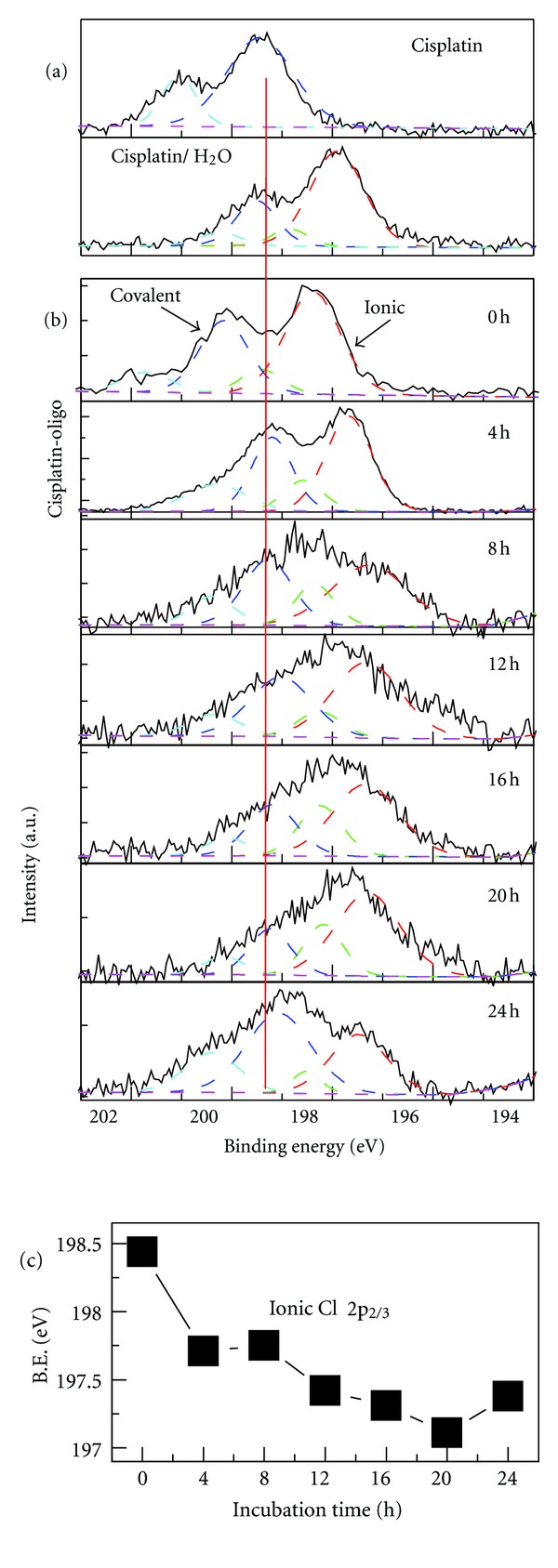
(a) High-resolution XPS spectra of Cl 2p for cisplatin in solid phase and in H_2_O. (b) Cl 2p spectra for cisplatin-oligo complex at ratio of 8 : 1 with the increasing incubation time. The peaks are deconvoluted to covalent and ionic Cl (two coupled dashed lines), respectively. The straight line is used to point out the relative position of covalent Cl. (c) The binding energy of ionic Cl 2p_3/2_ corresponding to (b) as a function of incubation time.

Generally, two chemical states of chlorine exist in any compound, that is, the covalent-bound and the ionic states [39].  Figure 1(a) exhibits the core-level Cl 2p spectra of cisplatin in the solid phase (top curve) and in H_2_O (bottom curve). The individual Cl 2p peaks could be deconvoluted according to the chemical characteristics of chlorine. With the angular momentum coupling the 2p orbital of each type of chlorine consists of the spin-orbit-split doublet, that is, 2p_3/2_ and 2p_1/2_ [40]. Thus, the spectra of Cl 2p are curve fitted to two doublet subpeaks assigning to covalent (dashed upper curve in Figure 1(a)) and both covalent and ionic chlorine (dashed curves in lower Figure 1(a) and in Figure 1(b)). 

For cisplatin in the solid, without the perturbation of aquation all chlorine is expected to be covalently bound to Pt. In other words, the spectrum of solid state cisplatin represents the characteristic covalent Cl with BE of 199.6 and 201.1 eV (Figure 1(a)). When cisplatin is dissolved in H_2_O, it is known that the kinetic of the aquation process involves the replacement of Cl by H_2_O subsequently in two steps, resulting the coexistence of two types of Cl: ionic and covalent, around the bulk of cisplatin [25, 41]. The ionic Cl arises from the release of covalently bound chlorine from cisplatin and exists owing to the remaining electrostatic interaction, indicating that the BE of ionic chlorine is smaller than that of covalently bonded. This scenario is demonstrated in Figure 1(a) for cisplatin in water, where a strong signal of ionic Cl is observed and the covalent Cl signal remains. The latter is evidently seen with the identical BE as that of in solid cisplatin. The similar assignation of Cl is applied to the spectra of cisplatin-oligo complex. 

Figure 1(b) shows the core-level Cl 2p spectra of the cisplatin-oligo complex at molar ratio of 8 : 1 as a function of incubation time, ranging from 0 to 24 h at interval of 4 h. The high-resolution spectra of Cl 2p display dramatically different features with reaction time prolonging. At 0 h, which means that cisplatin is only mixed with oligos without the incubation at 37°C, two main peaks are observed corresponding to covalent and ionic Cl with characteristic BEs of 200.15 and 198.48 eV, respectively. The feature of the two main peaks remains after incubation for 4 h with the shift of related BEs to lower energies of 199.24 eV and 197.71 eV, respectively. With longer reaction time, the Cl spectra appear as a prominent broader peaks except at 24 h where the feature of the two peaks could be observed again to some extent. Applying the same principle of peak deconvolution, two types of Cl could be assigned in each spectrum with respect to various incubation times. The result clearly displayed that the BE of covalent Cl remained almost the same from 4 to 24 h, whereas the BE of ionic Cl decreased with a final relative increase of 0.27 eV at 24 h. The BE of ionic Cl as a function of incubation time is specified in Figure 1(c).

When cisplatin is mixed with oligos, the reaction channel of cisplatin directly interacting with oligos is expected to be competitive with the simultaneous aquation of cisplatin. In the process of hydrolysis, the chloride ions are progressively replaced by water (H_2_O) ligand, resulting in the production of cationic monofunctional and bifunctional adducts [41]. NMR studies have determined the half-time of cisplatin hydrolysis is *ca.* 2 h with *cis*-[Pt(NH_3_)_2_Cl(H_2_O)]^+^ as the dominant species which compromise *ca.* 8% of the total platinum complex within 2 h [41]. As revealed in Figure 1(a), the Cl spectrum of cisplatin-oligos at 0 h is similar to that of pure cisplatin, indicating a similar initial status of the reaction system. For pure cisplatin after 4 h of incubation the covalent component in the Cl spectra could be mainly assigned to the *cis*-[Pt(NH_3_)_2_Cl(H_2_O)]^+^ species with featured BE of 199.82 eV. Under identical reaction conditions, however, the Cl spectrum of the cisplatin-oligo complex varies substantially from the pure cisplatin with a pronounced BE shift of covalent-bound Cl (i.e., 199.18 eV versus 199.82 eV). Although it could not be accurately determined whether the formation of *cis*-Pt(NH_3_)_2_Cl-oligo adduct was due to the direct replacement of Cl ligand in cisplatin by oligos or replacement of water in *cis*-[Pt(NH_3_)_2_Cl(H_2_O)]^+^ by oligos, the significant difference of the spectra along with substantial BE shift simultaneously suggest that in the cisplatin-oligo system formation of *cis*-[Pt(NH_3_)_2_Cl (oligo)]^+^ adduct is the main pathway as compared with hydrolysis. In other words, cisplatin could react easily with oligos leading to irreversible DNA binding.

Notably, the full width at half-maximum (FWHM) for covalent and ionic Cl peaks at 4 h is approximately 1.0 and 1.1 eV, respectively. With the reaction time processing, the broadening of the Cl peaks, especially for the ionic Cl, is obvious in Figure 1(b). The enlargement of the peak reflects the increase of the inelastic electron scattering with the longer reaction time, which further demonstrates the formation of chemical bond between Pt and oligo bases as well as the release of ionic Cl. Evidence of ionic Cl release is reflected by the gradual decrease of BE for the ionic Cl 2p_3/2_ from 0 to 20 h (Figure 1(c)). The negative BE shift of ionic Cl indicates that Cl^−^ could be increasingly released with the binding of cisplatin to oligo. Around 20 h, it is expected that nearly all Cl ligands of the Pt species are replaced by oligo forming the *cis*-Pt(NH_3_)_2_(oligo) adducts such as *cis*-Pt(NH_3_)_2_GG, giving the major feature of ionic Cl in the spectra. At 24 h, the increase of the Cl^−^ BE and the recovered feature of two main peaks with characteristic BEs of 199.47 eV and 197.38 eV suggests that the interaction of cisplatin with oligos stabilizes around the incubation time of 20 h. Another explanation of the two-peak reappearance is the possible formation of bridged dinuclear platinum adducts, as reported by Davies et al. [41]. They reported that indication of the formation of bridged dinuclear platinum adducts is around 26 h [41].

The present result of high-resolution Cl spectra as a function of reaction time is consistent with the well-known cisplatin-DNA reaction schemes. In addition, it discloses that the process of cisplatin binding to DNA at a temperature of 37°C over a time scale of 24 h is more kinetically than thermodynamically controlled. Compared to previous studies focused on the Pt bonding, the signal of Cl by XPS technique could be a sensitive marker to directly reflect the dynamics of cisplatin reaction with DNA. 

### 3.2. The Effect of the Molar Ratio of Cisplatin to Oligo

**Figure 2:**
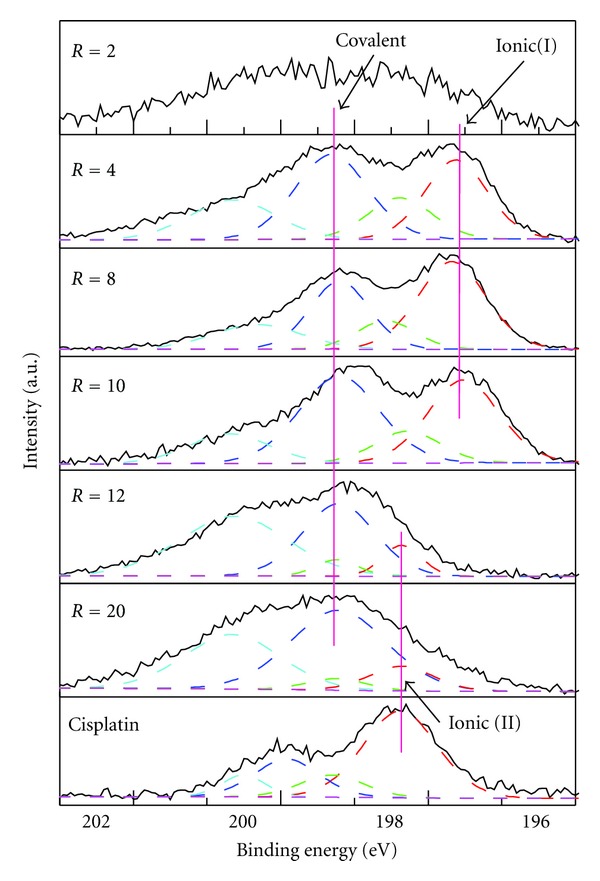
High-resolution XPS spectra of Cl 2p for cisplatin-oligo complexes with ratios (*R*) from 2 to 20 with identical amount (3.1 *μ*g) of oligo. All samples are taken after incubation at 37°C for 4 hrs. The three straight lines are used to assign the position of covalent and ionic (I) Cl bond position of cisplatin-oligo complexes as well as the ionic (II) Cl of cisplatin, respectively.

The Cl spectra of the complexes with various cisplatin/oligo ratios are illustrated in Figure 2 together with the pure cisplatin under the identical incubation time of 4 h. For better comparison, the amount of oligos was kept the same in the measurement; that is, only the amount of cisplatin increases. Accordingly, the amount of cisplatin with ratio of 2 is equal to 1 nmol, which is close to the detection limit of the XPS signal thereby leading to a broad Cl peak with relatively high signal-to-noise. Thus, with the exception of the ratio of 2 each Cl spectrum could be deconvoluted into two doublet 2p peaks assigned to covalent and ionic Cl, respectively (Figure 2). The Cl spectra of cisplatin-oligo complexes with ratios of 4, 8, and 10 exhibit similarly two main 2p_3/2_ peaks with BE of 199.2 and 197.7 ± 0.1 eV for covalent and ionic Cl, respectively. The assignment is consistent with the previous studies on the Cl containing compounds [42–51]. Moreover, the integrated peak area of each type of Cl indicated a relative percentage of ionic to covalent Cl of approximately 50% ± 10% for ratios of 4, 8, and 10. 

When the ratio increased to 12 and 20, a broader peak with higher BE is displayed. The result of reaction time has indicated that the BE of ionic Cl to the oligo is around 197.7 eV or lower. Compared to the spectra of cisplatin-oligos with lower ratios, it is surprising to find that the peak which could be resolved to similar ionic Cl (ionic I) does not exist in the cisplatin-oligo complex with ratios of 12 and 20. With such high BE, the broad peaks of cisplatin-oligo complex with ratios of 12 and 20 could only be assigned to covalent bond Cl. This assignment seems contrary to the previous result, indicating that cisplatin could react easily with oligos leading to the release of ionic Cl. How can we understand the apparent disappearance of the ionic Cl in Figure 2?

Noteworthily, the 20-base oligo (C**G**T**G**ACA**G**TTATT**G**CA**GG**C**G**) used in this study has 11 purines (G, A) indicating there are a total of 11 interaction sites for one cisplatin. Thus, in the case of mole ratio of cisplatin to oligo lower than 11, cisplatin could react completely, that is, binding to one of G or A bases and release Cl^−^ as indicated in Figure 1. When the ratio increases beyond 11, there is an oversupply of cisplatin with respect to the reaction sites. In other words, the system contains more cisplatin which cannot react with oligos and has to follow the aquation process. The increase in the amount of cisplatin results in the change of chemical environment in the cisplatin-oligo complex, leading to shift of the Cl spectra to higher BE. In fact, the Cl spectrum of pure cisplatin under identical conditions is resolved to covalent and ionic Cl with BE of 199.82 and 198.37 eV, respectively. Thereby, it is reasonable to deconvolute the spectra of Cl with ratio of 12 and 20 to the ionic Cl in pure cisplatin, as ionic (II) Cl shown in Figure 2. Moreover, multiple reactions could also occur in the system with ratios of 12 and 20 which is demonstrated by the broader peaks of Cl spectra compared to that of ratios lower than 11.

Determination of the platination of DNA with Pt-based drugs is usually confined to complicated inductively coupled plasma mass spectroscopy (ICPMS) which may not provide sufficient information on the specific interaction process [52]. Our results strongly evince that peak intensity and detailed chemical compositions deconvoluted from the Cl spectra are well correlated with the ratio of cisplatin to oligo. The covalently bound chlorine (−Cl) and ionic chloride (Cl^−^) species in different reaction procedures and in the presence of varied molar ratios of cisplatin could be readily differentiated solely from the high-resolution Cl spectra. That is, the Cl XPS spectrum could exhibit distinct features at different molar ratio levels, thus, making it an alterative* in situ* approach to trace the platination process of DNA. It also suggested that the characterization of Cl by XPS could be applied to monitor the interaction of chemotherapeutic agent cisplatin with DNA.

### 3.3. Chemical Bond Transformation of DNA Induced by Cisplatin

**Figure 3:**
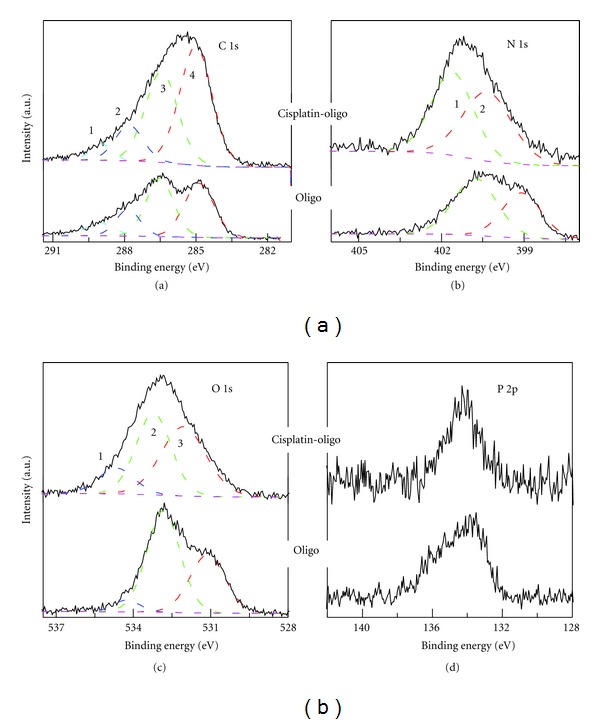
High-resolution XPS spectra of C 1s, N 1s, O 1s, and P 2p regions for pure oligos and cisplatin-oligo complexes at a ratio of 10 with the same amount of oligos in the films. The incubation time for the cisplatin-oligo complex is 4 hr. The peaks (solid curve) were deconvoluted into specific components of the oligo (dashed line) including (a) C 1s, urea (peak 1), amide (2), C–N/C–O–C/C–OH/N=C–C (3), and hydrocarbon (4); (b) N 1s, amino (1) and imino (2); (c) O 1s, C–OH (1), C=O/N=O (2), and phosphate group (3); (d) P 2p, the phosphate group. Note that the spectra in each figure are on the same scale, but offset for clarity.

In addition to the Cl spectrum, XPS could also be used to characterize the chemical bond transformations of DNA induced by cisplatin. The high-resolution spectra recorded for the four principal elements, C, N, O, and P of DNA (C 1s, N 1s, O 1s, and P 2p regions of cisplatin-oligo and oligo) are presented in Figure 3. A number of peaks are chosen to fit each elemental region corresponding to particular chemical bonding. The assignment of the spectral peaks is carried out according to the known substituent effects of core electron binding energies [53]. The XPS characterization of thymus DNA [54] and self-assembled monolayer DNA [55, 56] has also been reported previously. Due to structural similarity of oligo to DNA and for better comparison, we apply the same peak assignments referring to the work of Ptasińska et al. [54]. The specific chemical bond species relating to C, N, and O in the framework of oligonucleotide are displayed in the supporting information.

The C 1s spectra of cisplatin-oligo and oligo (Figure 3(a)) are curve-fitted to assign four types of carbon species: (1) urea [N–C(=O)–N], (2) amide (N–C=O), (3) alcohol/cyclic ether/carbon bond to nitrogen (C–OH/C–O–C/C–N/N=C–C), and (4) hydrocarbon (C–C/C–H). In the present study, the samples are sputtered with Ar ions before each measurement to obtain minimum carbon contamination in the XPS spectra. Thus, the C 1s spectra exhibit features uniquely due to the different contributions of carbon species in the bulk sample. The XPS C 1s spectrum of oligo shows faithful agreement with the previously reported single-strand DNA [55]. From the convoluted peak areas of the different carbon species, the percentage of corresponding 1–4 carbon components is found to change from 7.13%, 17.73%, 38.19%, and 36.95%, to 3.18%, 15.24%, 29.77%, and 51.18%, respectively, with the ratio of cisplatin to oligo increasing from 0 to 10. The constituent of C 1s spectrum varies appreciably for component 4 and the intensities greatly increase with the addition of cisplatin to oligo. It suggested that the binding of cisplatin may violently perturb the regular structure of oligonucleotides leading to the alternation of chemical environment. More specifically, with the inter- and intrastrand binding of cisplatin to DNA bases, greater effect of structural change, such as the perturbation of hydrogen bonding between bases, is expected to occur in the well-defined double-strand DNA.

The principal N 1s core-level peak consists of two-component structure for DNA with BE of 400.8 and 399.3 eV, respectively, consistent with published results [55]. The higher energy peak is attributed to amino N sites connected with single bonds, and the peak at the lower BE is assigned to imino species that include a double N=C bond. The N 1s spectrum of cisplatin-oligo displays different features in comparison with pure oligo: the increase of the intensity to a narrower peak and the shift of the peak to higher BE. It is known that cisplatin binds preferentially to the N7 sites of G or A yielding cisplatin-DNA adducts, and moreover, *in vitro *studies have shown that *cis*-Pt(NH_3_)_2_GG and *cis*-Pt(NH_3_)_2_AG intrastrand cross-link adducts account for 65% and 25%, respectively, in the total Pt-DNA species [57, 58]. Hence, it is expected that the peak 1 of N 1s for cisplatin-oligo includes the new contribution of the Pt–N bond. Compared to oligos, BEs of the amino and imino for cisplatin-oligos increased to 401.8 and 400.1 eV, respectively, indicating the formation of stronger chemical bond.

The O 1s spectrum of oligo is consistent with the previous report, including two main peaks and a relatively small peak with BE of 534.3, 532.8, and 531.1 eV, respectively. The small peak is deconvoluted according to the procedure of Dinsmore and Lee and assigned to the C–OH bond [55]. Nevertheless, for oligos, the alcohol is the smallest component in all the O species, which is negligible as indicated in [55]. A relative symmetric peak is observed in the O 1s spectrum of cisplatin-oligos. Similarly, it can be deconvoluted to three peaks having BE of 534.5, 533, and 532 eV. One possible explanation in terms of the change of O 1s spectra is that cisplatin could also react with carbonyl group near the N7 sites of guanine, as suggested by Macquet and Theophanides [59], which may contribute to the change of carboxyl/carbamido (2) species. Furthermore, it is noticeable that peak 3, which is assigned to oxygen in the phosphate group, is substantially shifted to higher BE by 0.9 eV. Since there is no bonding of cisplatin to the backbone, it is speculated that cisplatin binding to DNA bases may exert influence on the chemical bond environment of sites neighboring the backbone. In the case of P 2p spectra, a peak with increased BE of 0.2 eV is observed for cisplatin-oligo. In this regard, we speculate that the binding of cisplatin may authentically affect the backbone in that P 2p spectra can only be attributed to the phosphate of backbone; this result is in agreement with the interpretation of the result of O 1s spectra.

To recapitulate, significant changes are clearly observed in the XPS survey spectra of cisplatin-oligo complexes compared to that of pure oligo (Figure S5). It was explicitly demonstrated that the BEs and peak intensity in oligos with regard to the C 1s, N 1s, O 1s orbitals were perturbed with the addition of cisplatin, a phenomenon which lies at the basis of the mechanism for the cytotoxicity of cisplatin in cell. 

### 3.4. DNA Chemical Bond Transformation during the Incubation Time

**Figure 4:**
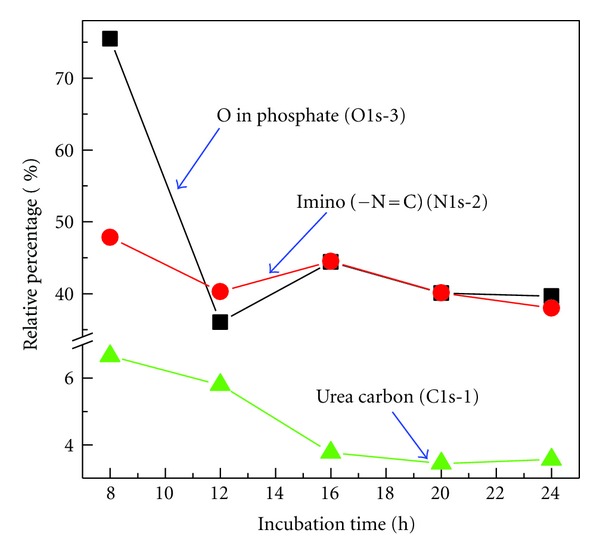
The relative percentage of O–P (■), N=C (●), and C–N (▲) bonds for a cisplatin-oligo complex with ratio of 10 deconvoluted from the corresponding O 1s, N 1s, and C 1s peaks as a function of incubation time. The percentages were obtained from a similar Gaussian deconvolution procedure as shown in Figure 3.

In addition to Cl spectra, chemical bond transformation during the incubation time could be related to the C, N, O, and P spectra. Applying the similar Gaussian curvefitting shown in Figure 3, the relative peak percentage of different components for C, N, and O species at various incubation times could be obtained. The relative percentage of O in phosphate (O 1s-3), N in imino (N 1s-2), and C in urea (C 1s-1) as a function of incubation time from 8 to 24 h is illustrated in Figure 4. The peak percentage of the typical three bonds of C, N, and O displays a similar trend, that is, decreasing dramatically from incubation time of 8 to 12 h along with relative saturation from 16 to 24 h. It indicates that no matter to what extent cisplatin binding influences the O, C, and N bonding in DNA, the chemical bond is more stable with the incubation time longer than 16 h. Thus, to get better equilibrium of chemical reaction between cisplatin and DNA and obtain stable cisplatin-DNA complexes for further radiation study, we suggest an optimum incubation time for cisplatin of about 20 h. The result also implies that for concomitant chemoradiation therapy involved Pt-based drugs the optimum uptake of cisplatin could occur within one day after injection.

## 4. Conclusion

The dynamics of cisplatin interaction with a 20-mer oligo have been systematically monitored by XPS technique. High-resolution XPS spectra of Cl in cisplatin-oligo complexes showed characteristic features with respect to different reaction time and ratios of cisplatin to oligo. The result indicated that characteristic Cl signal obtained by XPS could be employed as a sensitive marker to disclose the reaction dynamics of cisplatin binding with DNA, which at 37°C mimics the process in chemotherapy. By accurate measuring the spectra of other principal compositional elements of oligo (i.e., C, N, O, and P), the shift of the BE as well as peak intensity with the binding of cisplatin were, for the first time, observed. Since the structural and chemical bond modifications may provide information related to the cytotoxicity of cisplatin in cell, our results would point to promising vistas on XPS as a novel characterization platform to investigate the dynamics of specific processes in the reaction of cisplatin with DNA, along with complementary results from traditional methods. In fact, the technique may have widespread applications to monitor the reaction dynamics of other Pt-based chemotherapeutic agents, such as carboplatin and oxaliplatin. Recently, engineering of cisplatin nanoparticles with glycol-functionalized copolymer exhibit improved antitumor efficacy [60], indicating the continuous opening of the development of novel cisplatin drugs. Further systematic work along this line on interaction between Pt-anticancer drugs and DNA is in progress in our lab.

## Supplementary Material

Figure S1: displays the high-resolution P2p spectra of cisplatin-oligo complex with ratio of 10:1 before and after Ar etching. It shows that the binding energy (BE) and the intensity of P2p peaks remain the same after applying Ar sputtering on the characterized spot. Unlike the other elements, C, O, and N, which also exist in air, the signal of P exclusively arise from the DNA phosphate backbone. Therefore, the only very slight change in the P2p spectrum shows that Ar etching under the present conditions does not affect the chemical composition of oligo and oligo-cisplatin complexes.
Figures S2-S5: shows the C1s, N1s, and O1s spectra of the oligos. The peaks are deconvoluted according to the different chemical bonds of each element in the oligo. For clarity, the specific chemical bonds are pointed out in the structure of the cisplatin-oligo complex and illustrated in the same figure.
