# Supplementary material for: Sensitive Marker of the Cisplatin-DNA Interaction: X-Ray Photoelectron Spectroscopy of CL
Source: Bioinorg Chem Appl. 2012 Oct 24;2012:649640. doi: 10.1155/2012/649640 (PMC3485869; doi:10.1155/2012/649640)
Supplement: Supplementary file 1 — Figure S1: displays the high-resolution P2p spectra of cisplatin-oligo complex with ratio of 10:1 before and after Ar etching. It shows that the binding energy (BE) and the intensity of P2p peaks remain the same after applying Ar sputtering on the characterized spot. Unlike the other elements, C, O, and N, which also exist in air, the signal of P exclusively arise from the DNA phosphate backbone. Therefore, the only very slight change in the P2p spectrum shows that Ar etching under the present conditions does not affect the chemical composition of oligo and oligo-cisplatin complexes. Figures S2-S5: shows the C1s, N1s, and O1s spectra of the oligos. The peaks are deconvoluted according to the different chemical bonds of each element in the oligo. For clarity, the specific chemical bonds are pointed out in the structure of the cisplatin-oligo complex and illustrated in the same figure. [file 649640.f1.pdf]

Supporting Information for the Manuscript

Sensitive Marker of the Cisplatin- DNA Interaction: X-ray Photoelectron Spectroscopy of Cl

Fangxing Xiao, Xiaobin Yao, Qianhong Bao, Danzhen Li, Yi Zheng*

Research Institute of Photocatalysis, State Key Laboratory Breeding Base of Photocatalysis, College of Chemistry and Chemical Engineering, Fuzhou University, Fuzhou 350002, PR China.

RECEIVED DATE:

TITLE RUNNING HEAD: cisplatin interaction with DNA

*CORRESPONDING AUTHOR:

Research Institute of Photocatalysis, Fuzhou University, Fuzhou 35002, PR China.

Telephone: (86) 0591-83779153

Fax: (86) 591-83779105

Email address: Yizheng@fzu.edu.cn

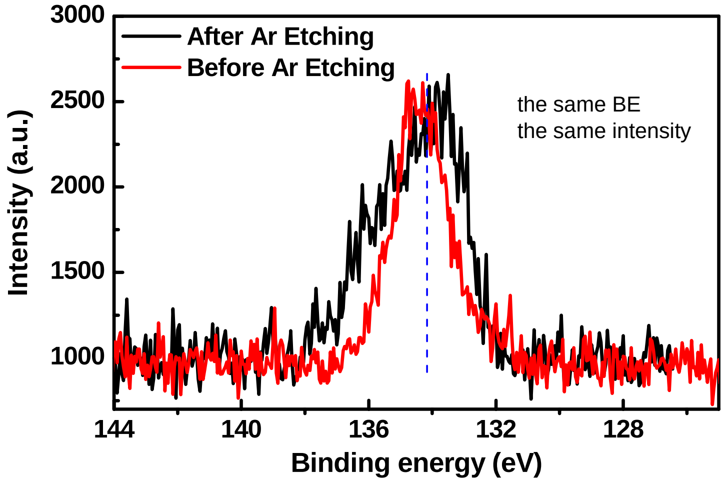

Fig. S1 The P 2p spectrum of cisplatin-oligo complex with ratio of 10:1 before and after Ar etching.

➢ **Carbon**[1]

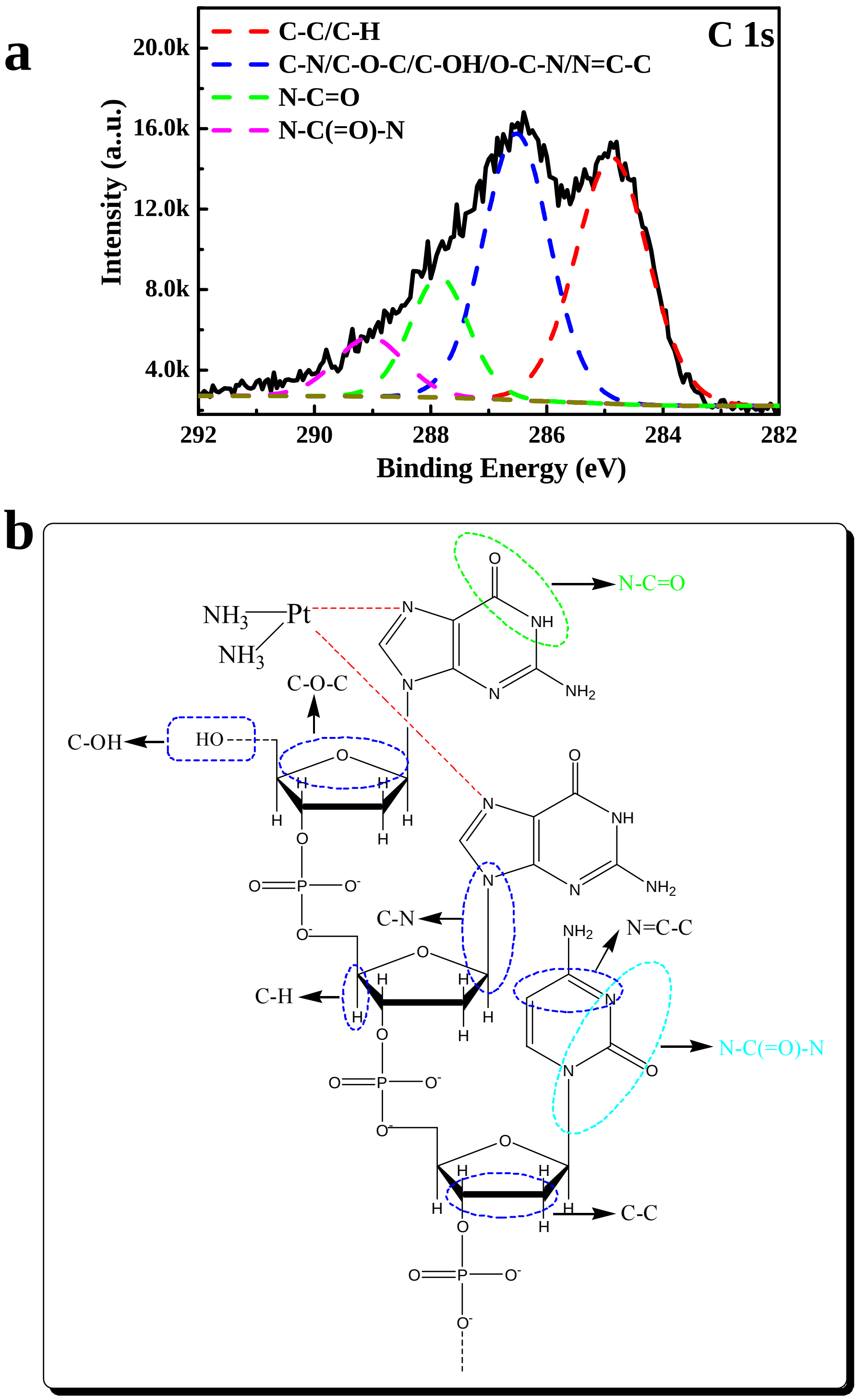

Figure S2. (a) High-resolution XPS C 1s spectrum of pure oligo and (b) schematic illustration showing the detailed chemical bonds species in the framework of cisplatin-oligonucleotide complex.

➢ **Nitrogen**[2,3,4,5]

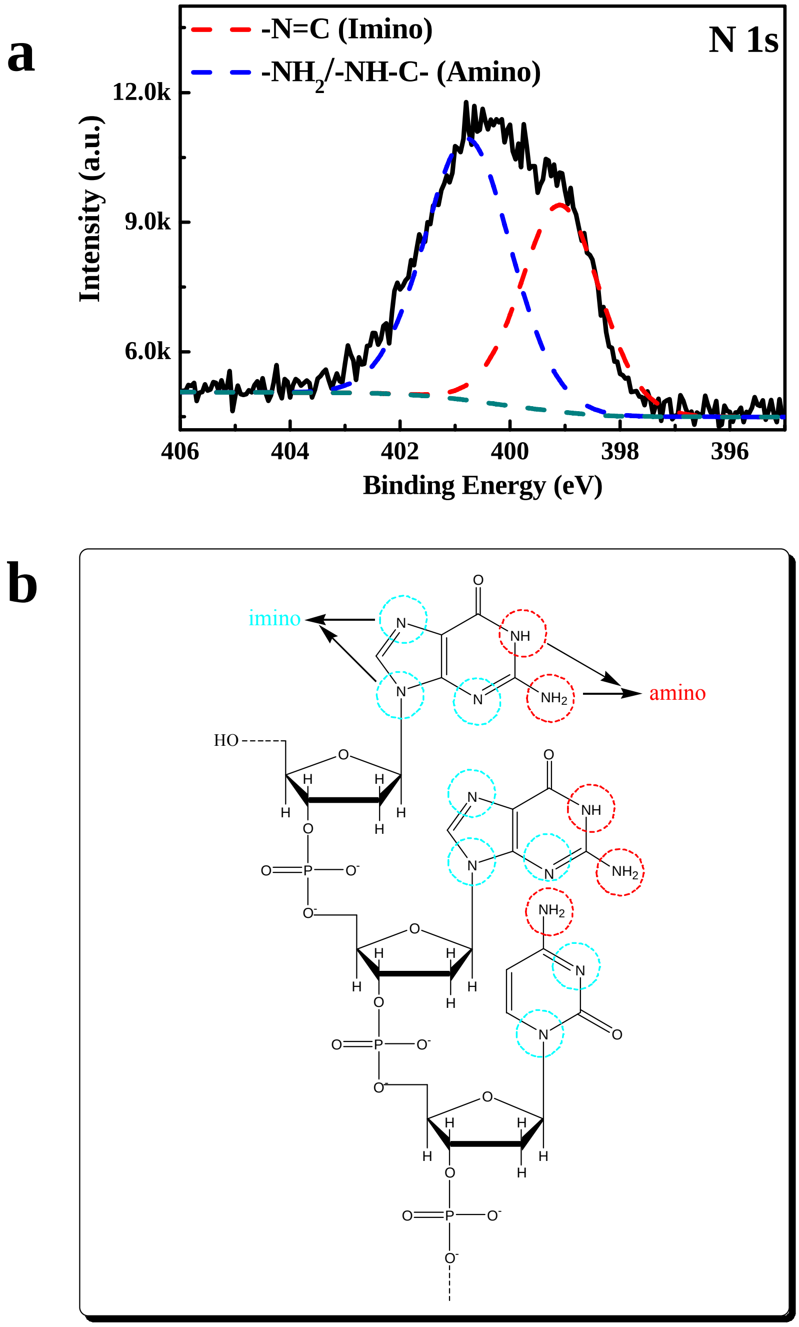

Figure S3. (a) High-resolution XPS N 1s spectrum of oligo and (b) schematic illustration presenting the specific chemical bonds species in the framework of cisplatin-oligonucleotide complex.

➢ Oxygen[1]

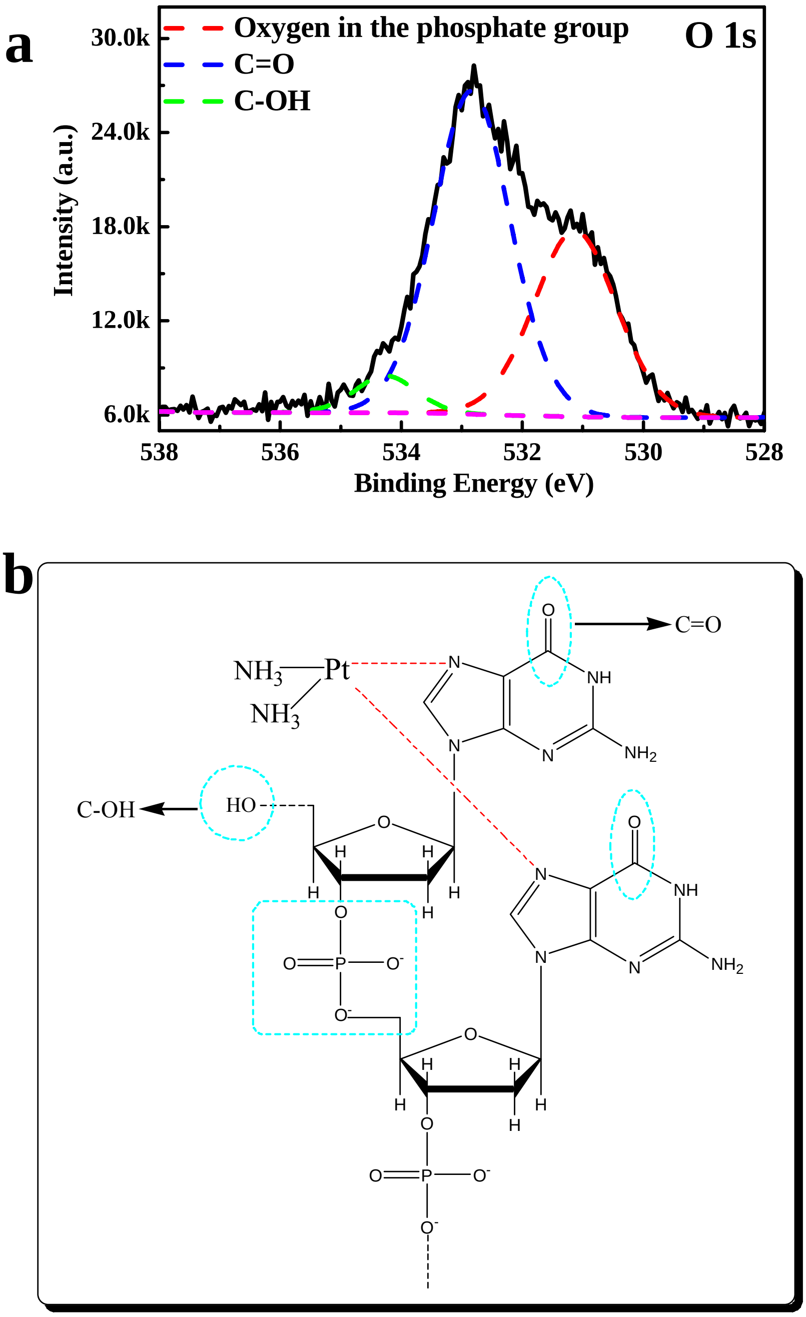

Figure S4. (a) High-resolution XPS O 1s spectrum of oligo and (b) schematic illustration showing the detailed chemical bonds in the cisplatin-oligonucleotide complex.

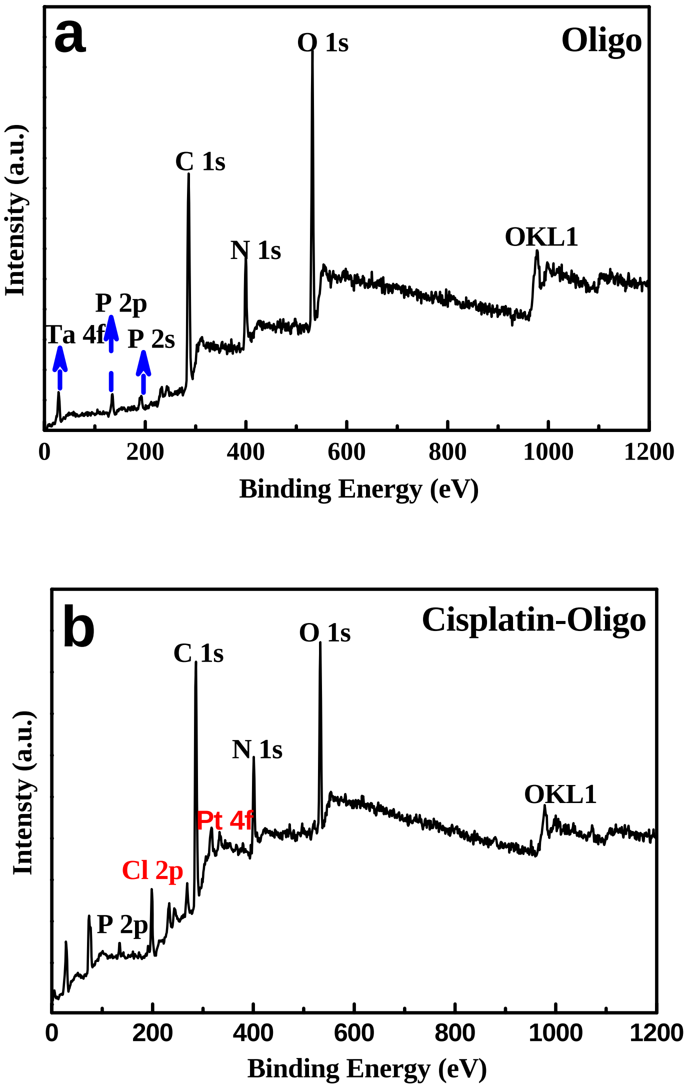

Figure S5. (a) XPS survey spectra of oligo and (b) cisplatin-oligo complex on the tantalum film.

➢ Reference

1 S. Ptasinska, A. Stypczyńska, T. Nixon, N.J. Mason, D.V. Klyachko, and L. Sanche, "X-ray induced damage in DNA monitored by X-ray photoelectron spectroscopy," *J. Chem. Phys.*, 129, 129-134, 2008.

2 E. Mateo-Marti, C. Pradier, and J. Martı´n-Gago, Ultraviolet Photostabilityof Adenine on Gold and Silicon Surfaces. *ASTROBIOLOGY*. 9, 573-579, 2009

3 E. Mateo-Marti, C. Briones, E. Roman, E. Briand, C.M. Pradier, and J.A. Martin-Gago, Self-assembled monolayersof peptide nucleic acids on gold surfaces: a spectroscopic study. *Langmuir* 21, 9510–9517, 2005.

4 M. Furukawa, T. Yamada, S. Katano, M. Hawai, H. Ogasawara, and A. Nilsson, Geometrical characterization of adenine and guanine on Cu(110) by NEXAFS, XPS and DFT calculation. *Surf. Sci.* 601, 5433–5440, 2007.

5 J. Magulick, M.M. Beerbom, and R. Schlaf, Investigation of adenine, uracil, and ribose phosphate thin films prepared by electrospray in vacuum deposition using photoemission spectroscopy. *Thin Solid Films* 516, 2396–2400, 2008.
